# Supporting the mental health and wellbeing of mothers at risk of repeat care proceedings and preventing care entry: a realist evaluation

**DOI:** 10.1016/j.chipro.2025.100166

**Published:** 2025-07

**Authors:** R. McGovern, E. Geijer-Simpson, S. O'Keeffe, W. McGovern, S. McCarthy, M. Lhussier, A. Bate

**Affiliations:** a Population Health Sciences Institute, Newcastle University, Newcastle Upon Tyne, UK; b Department of Public Health and Caring Sciences, Uppsala University, Gothenburg, Sweden; c Faculty of Health and Life Sciences, Northumbria University, Newcastle Upon Tyne, UK

**Keywords:** Pregnant mothers, Infants, Repeat care proceedings, Mental health and wellbeing, Realist evaluation

## Abstract

**Background:**

The number of children being taken into care is increasing year-on-year around the world, with particular concern about mothers who experience repeat infant removal. These mothers have often experienced multiple disadvantages, which are compounded by the trauma of child removal. There is an urgent need to understand how to interrupt these cycles of disadvantage, trauma and child removal.

**Objective:**

To identify and explain the perceived influence of contextual factors and mechanisms of an intensive, relationship-based intervention upon: i) the mental health and wellbeing of mothers at risk of repeat care proceedings; ii) and the care outcomes of their infants.

**Participants and setting:**

The study was conducted in the North East of England. Participants of the were 159 mother-infant dyads (n = 79 intervention and n = 80 received usual care). Additionally, we recruited a total of 39 realist interview participants (n = 10 referring practitioners; n = 11; Startwell practitioners; n = 17 mothers and n = 1 partner).

**Methods:**

This mixed-method study employed a realist approach to service evaluation, using quasi-experimental and qualitative methods.

**Results:**

In iterative consultation with stakeholders, we developed three programme theories relating to: i) supporting the mother to safeguard the child; ii) safely challenging behaviour and beliefs; iii) grief and loss.

**Conclusion:**

We found that in the context of surveillance wherein mothers with longstanding negative and adversarial experiences of services, a mother-centred and empathic approach is likely to lead to more accurate and decisive assessment, increase reunification and support mothers to grieve following infant removal.

## Highlights

• We conducted a realist evaluation utilising quasi-experimental and qualitative methods.
• We examined what works to support the mental health and wellbeing of mothers and prevent care entry of their infants.
• Child safeguarding teams should separate supportive functions from safeguarding functions within the child protection plan.
• There should be a trauma-informed focus upon the mother's needs within plans which seek to safeguard the infant.
• Mothers should receive on-going support from a professional after infant removal.

## 1 Background

During the past decade there has been a year-on-year increase in the number of children who have entered care in England (Department for Education, 2023), the United States (US Department of Health and Human Services, 2019), and elsewhere around the world (Australian Institute of Health and Welfare, 2024). There is particular concern regarding the notable proportion of mothers who experience child removal and return as a respondent in successive care proceedings (Broadhurst et al., 2015; Jonson-Reid et al., 2019), growing recognition of the needs of fathers who experience recurrent proceedings (Bedston et al., 2019; Philip et al., 2020) and the rising number of ‘babies being born into care’ (Bilson & Bywaters, 2020; O'Donnell et al., 2019). The majority of the mothers who experience recurrent care proceedings have experienced multiple disadvantages throughout their lives (Cox, 2012). They are often considerably younger at the time of their first birth than both the general population of mothers and those mothers that experience a single care proceeding (Broadhurst & Mason, 2017). Many have experienced adversity in their childhoods which led to them being placed in out of home care themselves as children and they are typically exposed to additional risk factors in their adult life, such as domestic violence, substance use and mental health problems (Broadhurst et al., 2017). Further, indigenous and first nation children continue to be over-represented in care proceedings (Chamberlain et al., 2022; O'Donnell et al., 2019).

There is an urgent need to understand how to interrupt the cycles of trauma and child removal experienced by mothers who are subject to repeat care proceedings (Family Rights Group, 2018). The recent Independent Review of Children's Social Care in England found the child protection system to be focused upon assessment and crisis intervention, and called for better support for families to prevent care entry (MacAlister, 2022), with similar calls of reform worldwide (Chamberlain et al., 2022; Lindell et al., 2020; Wise, 2017). The need to protect children from harm is unquestioned, and where it is not safe for a child to remain with their birth parents, decisive and timely action is required (MacAlister, 2022). However, the removal of a child can compound the mother's vulnerability, creating both an immediate psychosocial crisis (Broadhurst & Mason, 2020); whilst also having an accumulative and enduring adverse impact (Broadhurst & Mason, 2017). The enormity of the recovery challenge following the removal of a child is increasingly recognised (Broadhurst & Mason, 2020). Alongside this is a growing acknowledgment of the moral and economic imperative to support the mental health and wellbeing of mothers who have experienced care proceedings and to affect change in circumstances to prevent recurrence (Broadhurst & Mason, 2013).

Over the past decade there has been a rapid growth in services for mothers at risk of recurrent care proceedings. However, there remains a paucity of evidence for interventions to prevent children being taken into care (Cox et al., 2020) and to support the mental health and wellbeing of these mothers (Easter et al., 2022). The small number of studies which have been conducted report positive effects, including a reduction in unplanned pregnancies and/or care entry (Cox et al., 2017, 2020; Jones et al., 2024; McCracken et al., 2012, 2017; Roberts et al., 2018). However, these studies have typically employed a single group pre and post design, and were therefore unable to attribute any changes to the intervention (Marsden & Torgerson, 2012). Whilst other outcome evaluations required participants to take a reversible contraceptive throughout the study period and may have delayed rather than prevented repeat care proceedings (McCracken et al., 2012, 2017). Moreover, studies do not consider the complexity within the intervention itself; how or why it works (or not). For example, a recent mixed method evaluation combined the outcomes of three separate services which the authors reported shared ‘core values’ (Cox et al., 2020). However, the properties of the intervention itself; the extent of tailoring to individual need; the range of behaviours or outcomes targeted and the expertise, skills and approach of those delivering the intervention were not considered. Such limitations within the current evidence-base reduces the usefulness of the evidence to decision-makers and falls short of providing the understanding necessary to develop adaptable or scalable programmes that work for all mothers. There is therefore an urgent need to build this evidence base to understand what works to support pregnant mothers at risk of recurrent care proceedings.

### 1.1 Current study

In July 2020, Barnardo's national charity in the UK developed the ‘Startwell’ service. This service received local government funding to be piloted in one local authority area in the North East of England and then was extended to five additional local authority areas in April 2022. The Startwell service provides an in-home intervention to pregnant mothers who have previously had one or more child removed from their care, and where there was a risk of care proceedings following the birth of their infant. The service is described as providing an integrated, intensive, and systemic model of support based on assertive outreach, relationship-based practice, which is informed by attachment theory, trauma, grief and loss and strength-based practice. Practitioners have a range of professional backgrounds including social workers, family support workers and parent trainers. The Startwell service aims to set in place foundations on which mothers can build a more positive future for themselves and keep their infant safe by understanding and consistently being able to prioritise their needs. They provide practical support to attend and engage with a wide range of health and social care services in the community, to ensure appropriate antenatal care and support is accessed and services are provided in response to the mother's health needs. Support is intended to begin at the earliest possible opportunity following pregnancy being confirmed and continues for around one-year, irrespective of whether the infant remains with the mother. Typically, the intervention duration is 18 months. Further information is available via the charity website (https://www.barnardos.org.uk/get-support/services/foundation-startwell). In this study, we examined the Startwell intervention with the aim of deriving explanatory evidence that describes what works to support the mental health and wellbeing of mothers at risk of recurrent care proceedings, and to prevent care entry of their children.

## 2 Methods

This mixed-method study employed a realist approach to service evaluation, using quasi-experimental and qualitative methods. Realist methodology unpacks and explains the possible causal and contextual factors of change and is therefore ideally suited to examining complex social interventions, whose influence is context-dependent (Pawson, 2006). It acknowledges that interventions take place within complex social systems and uses the formular of Context ​+ ​Mechanisms ​= ​Outcome (C ​+ ​M=O) to explore and test the programme theory underpinning interventions (Ray Pawson & Nick Tilley, 1997). Within this study, we have delineated mechanisms into two categories allowing us to separate and consider those which are resource mechanisms (i.e. the pragmatic intervention details) and those which are response mechanisms (the underpinning behaviour and/or affect triggered by the resource that helps explain the outcome) (Dalkin et al., 2015). The study was conducted in the three iterative phases of realist evaluation: i) develop initial programme theories; ii) test the programme theories using empirical data; and iii) refine the programme theories (R. Pawson & Tilley, 1997a, 1997b). We have followed the RAMESES II reporting standards for realist evaluation (Wong et al., 2016).

### 2.1 Phase 1: develop an initial programme theory

In this Phase we worked with a range of stakeholders (consisting of mothers with experience of the intervention, service managers within the charity, Startwell practitioners and social workers referring into the service) to identify the explicit and implicit assumptions about how the intervention ‘worked’. Through these discussions we were able to elicit and identify a range of initial contextual and mechanistic factors that were thought to influence the outcomes. We then examined these factors within theory gleaning interviews with Startwell practitioners and social workers referring into the service, resulting in six candidate programme theories which were presented as ‘if X, then Y’ structured statements (see Table 1 for further details).

**Table 1:** Candidate programme theories.

If, then statements
If support is provided to the mother by a practitioner who is not responsible for safeguarding the child, the mother may be better able to have her needs met.
If a practitioner provides consistent support which includes high frequency and intensity contact with a mother, then they will develop a trusting relationship.
If mothers trust the Startwell practitioner, they may be able to engage effectively with the intervention.
If the mother is provided with practical assistance and support to attend appointments with services, the health of the mother and the baby may improve.
If care is tailored to the mother's individual needs and helps them to navigate and access a range of health, welfare and issue-based services, the identified risk factors may be reduced.
If continuous care is provided by a consistent practitioner, the mother may be supported during key transitions.

### 2.2 Phase 2: testing the programme theory using empirical data

The candidate programme theories developed within phase 1 were tested through quantitative and qualitative data. In keeping with the progressive intent of realist syntheses (Pawson et al., 2005), we worked closely with the Startwell practitioners, service manager and senior managers within the charity, wherein we iteratively consulted these stakeholder to test and refine the theory throughout the project. Congruent with realist evaluation, the analysis was iterative and also enabled the emergence of new programme theories through the data; the analytical process was thus concurrently inductive and deductive.

### 2.3 Quasi experimental study

A quasi-experimental study was conducted using administrative child protection outcomes. Unlike traditional outcome evaluations, the use of such methods in realist evaluation is not driven by binary questions of effectiveness. Rather, our quasi-experimental study enabled us to test how mechanisms in context produce outcomes (in other words, to test our programme theories) (Hawkins, 2016). A formal sample size calculation for the quasi-experimental study was not conducted. The sample for the intervention group consisted of all pregnant mothers who had received the Startwell intervention and given birth between July 2020 and December 2023 in the five intervention sites. The comparison group was all pregnant mothers residing in a neighbouring local authority (within a single site); who were subject to a child protection plan; who had given birth during the same period; and had one or more previous care proceeding. Both groups were deemed at risk of care proceedings determined by the unborn child being in receipt of a child protection plan and consideration being given to a Care Order under section 31(a) of the Children Act 1989. The comparison site provided standard in-house child protection intervention, based upon the signs of safety model (Turnell & Edwards, 1999). Anonymised routine data was used to compare the care status of infants born to mothers receiving the Startwell intervention to those receiving usual care, at birth (defined as the first 7 days of life), aged 6 months and 12 months and included socio-demographic data relating to age and ethnicity of mother; date of registration; gestation of infant at start of intervention; category of abuse; Index of Multiple Deprivation (IMD) rank and decile. This study thus predominantly enabled us to clarify contexts and outcomes to develop and refine programme theories.

#### 2.3.1 Data analysis

Quantitative analysis was conducted in Stata. We used logistic regression to analyse the quasi-experimental data and inform theory development and refinement, with p values of <0.05 considered significant. Initially we examined causal inks between different dyads. The first regression looked at the likelihood of the infant being born into care. The second regression looked at the likelihood that an infant was in care at 6 months. The third regression looked at the likelihood that the infant was in care at 12 months. We identified that both being born into care and receiving Startwell created key contexts which interacted with the mechanisms. To take account of these structures (or context-mechanism-outcome configurations) thought to be important in producing outcomes (Hawkins, 2016), a fourth regression was performed, with both variables combined into a single categorical variable. Further, all four logistic regressions used category of abuse, IMD decile (categorised as 1, 2 or 3+ due to small numbers of mothers being in deciles 3 and above) and whether or not the mother was registered more than twelve weeks before the birth of the infant as independent variables. Bivariate analysis on the probability of being born into care was performed on relevant variables (category of abuse, IMD decile, being known for more than 12 weeks before birth, being born into care and receiving the Startwell programme). However, as all models used in the bivariate analysis showed a poor explainers of the variability within the data, with no model having a McFadden's pseudo R^2^ greater than 0, multivariate logistic regressions were used.

### 2.4 Realist interviews

We conducted realist interviews with mothers who were currently/had recently received the Startwell intervention, practitioners who referred mothers into the service, and Startwell practitioners between February 2023 and January 2024. To achieve recruitment to the study, the researchers attended a Startwell team meeting to explain the study and invite participation. Both referring practitioners and mothers were recruited through Startwell practitioners, who informed the potential participants about the study and requested consent to share their contact details with the research team. Researchers then contacted potential participants by telephone or email to provide the participation information sheet and explain the evaluation. Written informed consent was obtained and interviews were scheduled at a time convenient for participants.

Researchers followed a realist topic guide, which was iteratively refined and informed by on-going theory-development. Specifically, we commenced our data collection focused upon the six candidate programme theories identified in phase 1 (see Table 1), which we hypothesised were important in explaining how and why Startwell ‘worked’. Within phase 2, we sought to further examine these candidate theories to add conceptual depth, examining the contextual factors (mother's characteristics/service provision external to the Startwell intervention), mechanisms of change (a combination of resources offered by an intervention and the triggered response) and observable, measurable outcomes. The exploratory questioning about the Startwell intervention was designed to draw out the propositions of the general inquiry of what works within this context (Manzano, 2016). Interviews were conducted by telephone, Microsoft Teams or in-person, depending on participant preference and were audio-recorded and transcribed verbatim by a transcription service approved by Newcastle University. Participants received a £10 shopping voucher in recognition of their contribution to the study.

#### 2.4.1 Data analysis

Data generated from the qualitative methods were coded using Nvivo and iteratively analysed by the post-doctoral researchers on the project (SO, EGS) using a realist framework. Explanatory statements (programme theories in the form of Context-Mechanism-Outcome configurations) from phase 1 of the study and which emerged inductively from phase 2 interview data, were empirically tested with the findings of the quasi-experimental study, and then served as the unit of analysis for further transcripts in an iterative process. The candidate programme theories were initially discussed and refined within the research team to identify where there was sufficient context-mechanisms-outcome support within the extracted data (Gilmore et al., 2019). In keeping with the progressive intent of realist syntheses (Pawson et al., 2005), we convened a theory-driven stakeholder workshop wherein we presented our refined programme theory and co-constructed chains of inference to further develop Context-Mechanism-Outcome configurations (Pawson et al., 2005; Rycroft-Malone et al., 2012), with explicit reference to the underpinning sources of evidence. This was then followed by further data collection through realist interviews, analysis and stakeholder theory-driven consultation.

### 2.5 Phase 3: refinement of the programme theory

The study iteratively collected and synthesised evidence from the quasi-experimental study, realist interviews and stakeholder consultation to produce a middle range theory which explains intervention influences in terms of both underlying mechanisms and setting (Hetrick et al., 2017; Ray Pawson & Nick Tilley, 1997; Porter, 2015). Middle range theories are programme theories about how the mechanisms of an intervention work in a specific context to bring about certain outcomes. They are specific enough to clearly explain the phenomenon, and general enough to apply across cases of the same type. Findings from different sources were compared and contrasted to identify occasions of confirmation and contradiction, with a detailed record of the process documented. We used a combination of abductive reasoning to seek the most plausible explanation for the hypotheses, and retroduction to identify hidden causal forces that lie behind identified patterns (Wong et al., 2016). It is usual within realist studies to draw upon established theory within this phase. We identified sociological theories of surveillance (Bentham, 2010; Foucault, 1988, 1991; Haggerty & Ericson, 2000) and stigma (Goffman, 1959) as providing a helpful lens through which to view and refine our programme theory and used these theories to examine complexity within the context. In particular, we applied these theories when considering the interaction between the mother and the safeguarding context and how this contributed to the activation (or not) of the Startwell intervention mechanisms. We applied deductive reasoning to test the programme theories and presented our refined programme theories to stakeholders and posed questions where causal chains were weak (for example, why some mothers appeared to be able to benefit from support to address the risks identified in the child protection plan and others did not). This process led to a consolidated account of how the intervention works (or not), for whom, in which circumstances and why.

#### 2.5.1 Ethical considerations

Child removal is a highly sensitive topic and has the potential to cause distress. A number of important mitigations were implemented to manage this risk. Interviews did not discuss traumatic life events the participating mothers may have experienced and/or the risks identified within the child protection plan. Instead, interviews were focused upon mother's experience of support. The researcher explained the purpose of the study before seeking consent to participate. Interview questions were provided in advance of the interview, at the request of the participant. Participating mothers were advised that they could choose not to answer a question(s) or could withdraw their consent at any time, without their care or rights being impacted. If preferred, participating mothers could be accompanied during the interview by a trusted individual. All participating mothers were provided with a debrief at the end of the interview. Whilst none of the participants reported experiencing distress during or following interview, our ethical procedures included alerting the Startwell practitioner to concerns for the mother's wellbeing so that they could provide support. The study was approved by the Faculty of Medical Sciences Research Ethics Committee, part of Newcastle University's Research Ethics Committee (Ref 2359/24340) This committee contains members who are internal to the Faculty. This study was reviewed by members of the committee, who must provide impartial advice and avoid significant conflicts of interests.

## 3 Findings

### 3.1 Participant characteristics

The quasi-experimental study included data from 159 infant-mother dyads; n = 79 received the Startwell intervention; n = 80 received usual care. The age of mothers across the sample ranged from 19 to 45 years (mean age 30.27 years), the majority of the sample were White UK (87.9 % of all where ethnicity is recorded) and lived in areas of high deprivation (78.6 % lived in an area amongst the 20 % most deprived). Most infants in the cohort were subject to a child protection plan due to neglect (characteristics of the participants of the quasi-experimental study are provided in Table 2). Results from the quasi-experimental study are integrated with the findings from the realist interviews to support the theory-driven analysis. A table detailing full findings of the logistical regressions is presented at the end of the paper (see Table 6).

**Table 2:** Participant characteristics.

Quasi-experimental study participant characteristic					
		Control		Intervention	
		n	%	N	%
N		80	100	79	100
	<21yrs	4	5	1	1.27
	21–30yrs	47	58.75	38	48.10
	31–40yrs	24	30	36	45.57
	40yrs+	5	6.25	4	5.06
Mothers age	Missing	0	0	0	0
	White UK	71	88.75	23	29.11
	Other	9	11.25	4	5.06
Ethnicity	Missing	0	0	52	65.82
Cat. Abuse/Neglect at Entry	Neglect	54	67.5	60	75.95
	Emotional	17	21.25	6	7.59
	Physical	7	8.75	2	2.53
	Sexual	2	2.5	0	0
	Missing	0	0	11	13.92
IMD Decile	1	48	60	43	54.43
	2	18	22.5	16	20.25
	3 +	14	17.5	13	16.46
	Missing	0	0	7	8.86
Registered more than or equal to 12 weeks before birth	No	37	46.25	36	45.57
	Yes	43	53.75	41	51.9
	Missing	0	0	2	2.53
Born into Care	No	48	60	26	32.91
	Yes	32	40	39	49.37
	Missing	0	0	14	17.72
Care aged 6 months	No	40	50	22	27.85
	Yes	37	46.25	28	35.44
	NA	3	3.75	14	17.72
	Missing	0	0	15	18.99
Care aged 12 months	No	42	52.5	30	37.97
	Yes	32	40	16	20.25
	NA	6	7.5	18	22.78
	Missing	0	0	15	18.99

Realist interview Practitioner characteristics	N = 21	%
**Gender**		
Female	19	91
Male	2	8
**Role**		
Social worker (referrer)	9	43
Midwife (referrer)	1	5
Startwell practitioner	9	43
Startwell Service Manager	1	5
Senior Manager – charitable organisation	1	5

Parent characteristic	N = 18	%
**Gender**		
Female	17	95
Male	1	5
**Age**		
<21 years	0	
21–25 years	2	11
26–30 years	4	22
31–35 years	6	33
36–40 years	5	28
41–45 years	1	6
**Ethnicity**		
White British	14	78
other ethnicity	4	22
**Number of previous child removals**		
1	3	17
2	6	33
3	3	17
4	5	28
5	0	0
6	1	6

We recruited a total of 39 realist interview participants, consisting of n = 10 referring practitioners (n = 9 social workers; n = 1 midwife), Startwell practitioners, service manager and administrator (n = 11), mothers who have received the Startwell intervention (n = 17) and a partner (n = 1) (characteristics of the participants of the realist interviews are provided in Table 1). Findings are presented below under three programme theories: i) supporting the mother to safeguard the child; ii) safely challenging behaviour and beliefs and iii) grief and loss. Supporting evidence as it relates to context, mechanism and outcome for each programme theory is presented in tables to illustrate the overall configuration, as opposed to individual findings. Due to the composition of the Startwell team (which included one male practitioner and one Service Manger), we do not report on the gender or specific role of the Startwell team member when providing verbatim quotation. This action was taken to maintain confidentiality.

### 3.2 Supporting the mother to safeguard the child

**Context-mechanism-outcome configuration:** Within a context of mothers having grown distrustful of services (context), the provision of non-adversarial support focused upon their needs and experiences (mechanism – resource) is perceived by the mother as safe, non-judgemental, trustworthy and empathic (mechanism – response) resulting in her engaging openly, an accurate assessment being made and timely response to safeguard the infant (outcome).

The primary focus of children's social care services is to safeguard the child. Whilst the necessity of prioritising child welfare is unquestioned, the interdependence between supporting the mother and the infant were at times overlooked by services and this led to the needs of the mothers being largely unconsidered and unsupported. Instead, these needs were framed within the child protection plan as risks *she* posed to her infant. Mothers often felt blamed for the difficulties they had encountered throughout their lives and perceived services as being adversarial and hostile towards them. This created a context for intervention wherein the mother was highly defensive and mistrusting of services, which were perceived as punitive, and were unable to openly discuss the difficulties they were experiencing, to ask for help, or to speak up if they were unsure what was expected of them. This was particularly evident in mothers who had experienced multiple care proceedings.

By having a separate service responsible for supporting the mother (Startwell), from that responsible for assessing the safeguarding needs of the child (children's social care services), these functions which could be perceived as potentially contradictory, are disentangled. The Startwell practitioner can focus upon the needs of the mother, asking ‘what *happened* to you’, rather than ‘what is *wrong* with your parenting?’ This trauma-informed approach was often perceived by mothers as compassionate and empathetic, resulting in mothers feeling understood and their experiences validated. The provision of each occasion of support in response to an existing need, along with the variability of needs the Startwell practitioner provided support for, gradually contributed to the mother lowering her defences, building trust, and engaging with the service (see Table 3).

Whilst mothers reported they were often guarded when discussing their circumstances with professionals involved in the child protection plan and will often choose to withhold or falsify information during interactions, the focus the Startwell practitioner has upon the mother's needs and experiences led to open and honest conversations about their situation. Mothers felt reassured that the information they shared would not be met with judgement about their ‘riskiness’ or prejudice about their character. Mothers highlighted that the Startwell practitioner was reliable and has genuine concern for the mother's wellbeing; characteristics that they cited as proof that the Startwell practitioners were *for* and not *against* the mother. Within the context of this relationship, Startwell practitioners were able to support mothers to attend antenatal appointments or access services in response to their own health needs, such as drug and alcohol services or mental health treatment; services which without the Startwell practitioners support, the mothers would have typically remained hidden from. This led to increased visibility of the mothers, their needs and ultimately the risk that may be present. As such, the involvement of Startwell practitioners were considered to be beneficial to accurate assessment of risk, resulting in timely and appropriate safeguarding measures. This is evidenced within the findings of the quasi-experimental study which showed that mothers who received the Startwell service were over two times as likely to have babies born into care (see Table 6; logistical regression 1; OR: 2.409; 95 % CI: 1.157 to 5.014; p = 0.019). Further, the duration of the relationship the mother had with the Startwell practitioner was directly associated with infants being born into care, wherein mothers who had been engaged with the Startwell practitioner for 12 weeks or more prior to the birth of their infant were more likely to experience removal at birth than mothers in the comparison group who were not provided with this service (see Table 6; logistical regression 1; OR: 2.090; 95 % CI: 1.020 to 4.283; p = 0.044). Mothers were supported to consider what they need, what their infant needs and the interdependence between their needs. In doing so, mothers come to recognise that care entry may be necessary and beneficial for the infant (Table 3).

**Table 3:** CMO configuration; Supporting the mother to safeguard the child.

Context	Mechanism	Outcome
*The way the system is, it's like they'd rather try to prove why you're not a good parent rather than put things into place like, “We're going to do this to help you (mother, 6 children removed from her care, participant 17).* *The welfare of the child is paramount, but the welfare of the child is dependent on what's going on for the parent. And that often gets ignored (Startwell practitioner, participant 106, interview)* *Sometimes they [mothers] want someone who's going to be on their side or they feel that you're on their side and I think you can do that up to a point, but then eventually, you can't be on their side, ‘cos you're effectively like, working in the best interests of the child … and I think that's a bit of a hard square to circle sometimes, because then they feel like you're not kind of … you're not trying to support them maybe as much as they'd want you to (male social worker, participant 208).*	*Like tag-teaming, so you have the social workers who have all the statutory responsibility … to the woman's viewpoint it's heavy-handed because they're the ones that are doing all the legal interventions around the children, they're doing the parent assessments, they're telling the woman what it is that they need to do to be able to keep the children. We can, kind of, tag team that so we can support the woman, help them to understand why children's services are saying the things that they're saying, help them to recognise what it is about their behaviours that they might need to change and why they might need to change them … (Startwell practitioner, participant 105)* *It's about when there's a barrier breaking the barrier down slowly; it's not about hitting it with a bulldozer, it's about right this barrier's in place for a reason, and respecting that enough and building up trust, I think, I think a lot of these women feel very, very let down … When I first met her, after an hour I could feel there was, there was a significant wall between us and I sat there for another hour and that hour, an hour extra made a massive difference cos she said I was the first person to properly listen to her. And, and I said ‘I hear you’, because that, you know they've got so many awful emotions and it's what they do with them. (Startwell practitioner participant 104, focus group)* *She [Startwell practitioner] listens to uz, she doesn't judge us and she understands like, sometimes where I'm coming from, about how like social workers were wrong in the past and she understood that and … She lets me tell the full story; she doesn't like, let us just tell her little bits and bobs; she lets us tell her the full story about what's happened and things (mother, baby currently in her care, three previous children removed for her care, participant 05).* *But just having someone to take me to my appointments, heavily pregnant, with my swollen ankles … sitting in the car for an hour, 2 h long and she would sit there and be there, waiting for me, when I came out. I just thought that was a beautiful thing to do for someone, just having that little bit of support there … It meant the absolute world to me at the time (mother, baby in her care, two previous children removed, participant 10)*	*Meeting someone who I can finally trust, and 100 % trust, you know. There's no reason to have that niggle in the back of my head where you're like, “Oh, I should really trust her this much?” I've just got 100 % trust. I think everything else I've touched on, to be honest. But trust is a big thing for me, so, allowing someone access into my life … She's done everything and more she could, while supporting me and [son]. So, I'm eternally grateful for the service (mother, one infant reunified, two children in care, participant 14).* *They [Startwell practitioners] can have - they have really, really good conversations with parents, because there isn't that professional barrier. Whereas, there is with [social work] practitioners, so to speak. So, it's not that they get information and then they grass on them, or anything like that, but it's more around sometimes if parents aren't being truthful with us, they may be truthful with their [Startwell] worker.” (female social worker, participant 207).*

### 3.3 Safely challenging behaviour and beliefs

**Context-mechanism-outcome configuration:** Where the mother has adequate intellectual, social and emotional resources (context), the supportive practitioner is able to utilise their benevolent relationship to safely challenge behaviour and beliefs that present a risk to the infant's wellbeing (mechanism – resource). Mothers feel hopeful in their ability to achieve change and work towards addressing the risks identified within the child protection plan (mechanism – response). As a result, mothers are more likely to retain the care of their babies or be reunified with infants who have been removed at birth (outcome).

The development of a trusting relationship between the mother and the Startwell practitioner provided a haven of safety for the mother during times when she felt under threat. When a mother shared a safe and open relationship with their Startwell practitioner, she was often more able to ask for help if experiencing difficulties. This resulted in timely support for a presenting issue as well as the avoidance of further deterioration of their situation. For example, mothers described being able to disclose an episode of substance use to their practitioner and therefore being supported to quickly return to abstinence. Startwell practitioners were also able to communicate ‘difficult to hear’ messages in a manner that many mothers were willing to accept. Mothers reported that they perceived a sense of hope for themselves and were empowered by the encouragement of the Startwell practitioner who they perceived ‘wanted them to succeed’ and believed in their ability to do so. Consequently, they were well positioned to challenge the behaviours or beliefs of mothers, which were considered to pose a risk to the safety of the infant. Whilst challenging conversations of this nature may be difficult for other practitioners to conduct, the consistency of the supportive relationship throughout the process and the frequency of contact enabled repeat discussion, and the opportunity for deepening reflection by the mother. These mechanisms operating in context, supported the prevention of care entry within the first year of life. The findings of the quasi-experimental study showed that infants of mothers who received the Startwell intervention were less likely to be in care at 12 months post birth (see Table 6; logistical regression 3 OR: 0.377; 95 % CI: 0.143 to 0.994; p = 0.049).

In many instances, this support was not sufficient to prevent care entry altogether. When an infant was taken into care, the mother often perceived this to be an adversarial act where they were being ‘blamed for their pasts’ or ‘things done to them’. Social workers (and other child protection care team members) were often criticised by mothers for their lack of understanding or consideration for how these experiences contributed to the difficulties experienced currently by the mother. As a result, many mothers would disengage from services following a decision to remove an infant and enter a period of psychosocial crisis, which made it less likely that they are able to achieve the change required to alleviate risk of harm to the infant. Indeed, having an infant born into care was highly predictive of an infant being in care at 12 months (Table 6; logistical regression 4; OR: 10.685; 95 % CI: 3.963 to 28.806; p = 0.000). However, we observed that the Startwell practitioner was able to maintain the mother's engagement following removal. Having demonstrated a concern for mothers, Startwell practitioners were thought to understand what had *happened* to mothers and therefore, *why* they behaved in particular ways, or held certain beliefs. This provided the optimum context for mothers whose infants had entered care to be supported through the initial crisis and then work towards reunification. When compared against infants who were *not* born into care and their mother's had *not* received the Startwell intervention, infants who *were* born into care and their mother did *not* receive the Startwell intervention were 15 times more likely to be in care at 12 months (Table 6; logistical regression 4; OR: 14.820; 95 % CI: 4.325 to 50.784; p = 0.000). Whilst those infants who *were* born into care and that *received* the Startwell intervention were almost 4 times as likely to be in care at twelve months (Table 6; logistical regression 4; OR: 3.951; 95 % CI: 1.293 to 12.014; p = 0.016).

Not all mothers were able to address the child protection concerns within their situation. Whilst the safety of the relationship enabled honest conversations to occur, some of the mothers struggled to comprehend the risks that were highlighted to them. Typically, the mothers who the Startwell intervention did *not* ‘work’ for, were mothers who continued to struggle to understand safeguarding processes and concerns after explanation from the Startwell practitioner. These mothers often had mild or moderate learning disabilities, demonstrated lower levels of reflective functioning, and were reluctant or unable to accept that their past behaviours and risks were relevant to the current child protection plan. They often lacked positive social support (for example supportive friends or family) and therefore the personal and social resources to facilitate change, making the scale of change appear unachievable. These mothers were most likely to continue to demonstrate behaviours or beliefs that were deemed a risk and were therefore least likely to retain the care of their baby or be reunified following a period of care (Table 4).

**Table 4:** CMO configuration: Safely challenging behaviour and beliefs.

Context	Mechanism	Outcome
*What we know is that they can't make good decisions, they are very vulnerable, we know statistically, to get back in another relationship that is violent … I've had, mums who are with violent partners have gone on to be with another violent partner in the future, I don't know how these people seem to find them, but they do. And it just kind of preparing them and give them the toolkit for what to look for, show them how to do Claire's Law, and I think sometimes it kind of goes in one ear and out the other (female social worker, participant 204).*	*The only way it was going to change [baby return to mother's care] was if mum would engage with really intensive therapeutic work … So, we knew she needed that additional support, no matter which way this case ended (female social worker, participant 206).* *I do whatever, deal with whatever comes along really because my, the women I work with, they just seem to have a multitude of things happens along the way, and, and it's about keeping them on the tracks a bit, because I think they will be easily derailed (Startwell practitioner, interview, participant 121).* *I think it was she had plenty of time to listen to me, to listen to how I felt. And when she would listen, she would not only listen to what I was saying, but she would challenge what I was saying, to get other things out of me about how I felt, why I felt like that. Was there anything different that I could have done that would have made me feel different? There was so much challenge from her, that gave her so many angles for me to open up to in ways that I never thought I would be able to (mother, five children in care, participant 05)* *I describe my job as, I feel, being a good parent. Because you do, you nurture, you support, you listen, all the rest of it. You advocate, but you can also kick them up the bum when they need it as well. So, that's where the honesty comes in (Startwell practitioner, focus group, participant 103).* *You don't feel alone, you feel supported and … Yes, it's like a nice feeling, it helps you feel a little bit stronger. There've been times when I've thought, “This is just so hard.” They'll give you a boost and … Yes, so it just makes you feel a lot stronger. They, kind of like, give you a bit of strength (mother, six children removed from her care, participant 16).*	*If it wasn't for [Startwell practitioner] helping me through it, I wouldn't- because social services were involved because of domestic violence and drug abuse, I think I would've relapsed, with the amount of stress it would've caused, if I'd tried to do it on my own, but she really helped me (mother, one infant reunified, four children in care, participant 07).* *We talked a lot about relationships. I didn't realise that they were abusive, you get used to anything and I'd seen a lot growing up. I wouldn't put up with it now. I know what to look for and I have the voices on my shoulders watching out for signs (mother, 3 children in care, participant 10).*

### 3.4 Grief and loss

**Context-mechanism-outcome configuration:** Where an infant is permanently removed from the care of the mother (context), practitioners with an enduring relationship with the mother can facilitate the mother's involvement in processes and activities relating to their infant (mechanism - resource). The mother experiences this as validating (mechanism – response) and they are able to begin to grieve the loss of their infant (outcome).

The removal of a child, particularly in the early days of life, is a highly distressing experience for mothers. The grief and loss they experienced was highlighted throughout the interviews and stakeholder consultation. It was recognised that whilst the experience of loss in these circumstances is largely unavoidable, the process itself was thought to be very difficult for mothers and often served to reinforce their trauma. The processes involved in child removal often included in-depth discussion about the parenting deficits of the mother, risk-taking behaviours and previous traumatic experiences, and as such served to induce feelings of shame, stigma and distress.

Mothers had typically found previous care proceedings to be a lonely and isolating experience and often contrasted these experiences to the valued support they were receiving from the Startwell practitioner in current care proceedings. There was evidence of practical and emotional barriers to providing and/or accessing support from other services during and after care proceedings, particularly in situations where permanency is achieved through adoption. Many of the services that had been involved during pregnancy, or whilst the infant was in the care of the mother, may no longer have a legitimate role and are therefore withdrawn. Mothers often feel that their identity as a mother is not recognised; that their grief and loss is somehow not valid. This often led to disfranchised grief wherein mothers feel inhibited from grieving due to the sense of shame and blame surrounding their loss. Conversely, mothers themselves may withdraw from services, particularly in circumstances where relationships are negatively affected, or where mothers hold these professionals responsible for the decision to remove the infant. The Startwell service was distinct from other services in that the legitimacy of involvement with the mothers remained irrespective of the care status of the infant. Moreover, the strength of the relationship between the Startwell practitioner and the mother that was formed out of non-adversarial support typically endured, with most mothers continuing to engage throughout. When there is no plan for reunification, the support provided by the Startwell practitioner shifts away from one of supporting the mother to safeguard the child, towards supporting her with the impact of removal. The Startwell intervention was therefore thought to be a critical source of support to reduce the trauma for mothers where a decision had been taken to place the infant in care.

Mothers mostly took comfort in their relationship with the Startwell practitioner, who supported them with their loss. They facilitated mothers to engage with activities which may promote their grieving process. For example, supporting mothers to spend time with their infant after birth was considered critical in responding compassionately to the grief they were experiencing at that time. Further, mothers were aided in tasks that maintained their identity as a mother beyond the decision to remove the infant. Specific examples included mothers attending meetings where care arrangements were discussed and contributing details about the pregnancy and birth to life story books so that the child's own sense of their identity maybe supported. Whilst involvement in these activities were considered important for the child's future wellbeing, they may also serve as acknowledgment of the significance of the mother's role, and therefore the significance of their loss. Supported exposure to these activities was thought to be helpful in enabling the mother to recognise that care was necessary and begin to reconstruct their parenting identity in the face of care entry. In doing so, mothers may reconceptualise their grief as having meaning, even benefit, and begin a gradual process of mourning and acclimation to their loss (Table 5).

**Table 5:** CMO configuration; Grief and loss.

Context	Mechanism	Outcome
*I think when you talk to [mothers], it just feels like it was something just done to them – they didn't have a voice that, nobody cared, nobody valued them, nobody cared about them nobody recognised that for them losing this baby was going to be like the end of the world, and if it felt like that to them, they felt like that and that they didn't want to live anymore (Startwell Worker, participant 122, interview).* *She [social worker] just dumped uz. You can't expect to take a baby off somebody whose carried that baby for*9 months *and then just dump her (mother, five children in care, participant 17).* *Normally when you kind of say right your baby's not coming home, this is the plan, we get no engagement, no phone calls are answered, they don't come to meetings anymore … normally I know I wouldn't probably see her again, even if I tried (female social worker, participant 204).*	*[The Startwell practitioner] was the one who actually went and supervised some of her family time at hospital when she'd given birth and she was really appreciative of that, erm, and it's just someone that she felt comfortable, who was able to always see that in a really difficult time in her life, when the baby was sadly not going to be staying with the young mother. But yeah, she was really thankful; that [Startwell practitioner] was able to be there and support her in that tough time (female social worker, participant 202).* *For some, you know, the babies won't go home, but at least if they can be engaged in that process and have their voice heard and have their say, it may not be the outcome that they really want to happen, but it means that in years to come, if that child as well does look at their records they'll see that mam's attended that meeting or gone to that contact. I think for me, that's another big part of it, you know, I have worked family support, I've worked with kids. I've worked with women that have been in the care system and, you know, wanted to request their records, social care records, and hearing that their mam didn't want them and wouldn't attend the contact wouldn't do this wouldn't do that - that has had huge implications on them. (Startwell practitioner, Participant 107, interview)* *For her [mother] to come back in and sit in front of me and do a life story book for her son, knowing that he's with adoptive parents … because of that [Startwell intervention] package of support. So, yes, it's not the outcome she wanted. It was the outcome the local authority recommended. But actually with the support that she was offered, she was able to see that she wasn't able to make the change required to keep her son safe. I don't think that would've been as well received had she not had the support from her Startwell worker (female social worker, participant 209)*	*I think it's reducing the trauma. Because the women are going through the process by themselves, because usually the other agencies have left them because it's focused around the child, and there hasn't been anybody to support the woman through that. Or go along with her, or sitting beside somebody whilst they're dealing with it, or talking it through, talking about how they felt about it. So, hopefully, it's reducing some of that trauma. It's hand-holding through the whole process. (Startwell practitioner, participant 104, focus group)* *One of the things that really got me was when a woman would tell you they understand that it was better for the baby not to be with them, that they knew they couldn't look after them at the moment. Your heart broke for the woman and all the things going on for her, but then still wanting to be the best mum and recognising what was best for baby. One of the most emotional was a woman who went to court and decided to withdraw her appeal against the adoption, even the judge was tearful as she explained that she thought it was in the child's best interests. She was broken afterwards, but we were able to keep working with her and ‘hold’ her while she went through the grief. (Startwell practitioner, participant 103, interview)*

## 4 Discussion

**Fig. 1:**
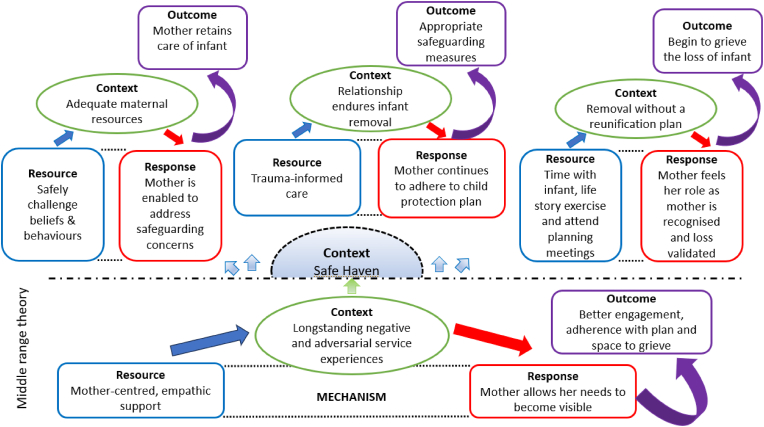
Programme theory.

In this study we used realist methodology to evaluate an intervention for pregnant mothers at risk of repeat care proceedings, examining mechanisms which are activated within this complex context to produce outcomes. We found that the Startwell intervention did not prevent infants being born into care. Indeed, those infants born to mothers who received the Startwell intervention and those that were known to Startwell for 12 weeks or more before birth, were more like to enter care in the first seven days of life. Combined, the findings of our realist evaluation suggest that the quality of the relationship between the Startwell practitioner and the mother allows the mother's needs to become visible resulting in accurate and decisive assessment. Given the complexity of need mothers at risk of recurrent care proceedings experience and the size of the recovery challenge, it may be unrealistic for a service initiated during pregnancy to prevent care entry altogether, and care at that time may in fact be in the best interests of the infant. However, in the context of surveillance wherein mothers have longstanding negative and adversarial experiences of services, a mother-centred and empathic approach shifts the locus of control and care, leading to better engagement, more proactive adherence with the child protection plan and space for healing and grieving. This provides mothers with the optimum opportunity to work towards reunification with their infant. This middle range theory is presented visually within Fig. 1; depicting the contexts in which pertinent mechanisms are activated to produce outcomes.

These findings can be best understood when observed through a sociological lens. Dominant theories of surveillance highlight the panopticon concept of ‘strategy of space’ wherein citizens experience a sense of constant surveillance (Bentham, 2010); enabling the few to watch the many. This notion, which is central to Foucauldian theory of power (Foucault, 1991), creates an invisible omnipresence of the observer resulting in discipline and compliance in the observed (Božovič, 2010). It follows that citizens subjected to panopticon forms of surveillance internalise this control, and associated morals and values (Foucault, 1980), for example of being a ‘good mother’ (Banwell & Bammer, 2006), resulting in the governing of the self (Foucault, 1988; Rose, 1999). Within child protection processes however, physical visibility is limited and therefore cannot account for the surveillance of mothers at risk of repeat care proceedings (Fong, 2020). Instead, state surveillance into private family life consists of the gathering of substantial information about marginalised and disadvantaged families from across systems (Barassi, 2020; Whittaker et al., 2020). Here, the many (services) observe the few (marginalised mothers). This ‘surveillance assemblage’, which is driven by a desire to bring systems together and integrate practices, exponentially increases the surveillance capacity (Haggerty & Ericson, 2000). However, the findings of our research add weight to the of others which show that mothers resist this surveillance (Fauci & Goodman, 2020). Under the threat of infant removal, mothers reduce their visibility to services through limiting disclosure (Gibson, 2015), or remaining hidden from services altogether (Broadhurst et al., 2017).

Mothers (like us all) engage in a ‘performance of self’ wherein they attempt to present a version of their self which they believe will be acceptable to the given audience (Goffman, 1959; Neale et al., 2011). When that audience is adversarial and focused upon the surveillance of risk, mothers seek to protect themselves from shame and stigma by performing their identity in a way which seeks to minimise or conceal risk. Moreover, mothers will also seek to conceal the struggles they encounter along the way (what Goffman referred to as the ‘dirty work’ of the self) (Goffman, 1959), presenting the ‘polished’, ‘finished version’. In doing so however, their needs are not identified, or met and their vulnerabilities (or risks as they may be perceived) increase. Herein, a discrepancy is often apparent between the performed and perceived self. Because of this discrepancy, mothers at risk of repeat care proceedings maybe viewed as both dishonest *and* risky; relationships between the mother and services may deteriorate and acts of adversarial intervention increase.

The findings of our study explain how to generate social interactions and practices within which, mothers at risk of repeat care can allow themselves to be visible to services and give a performance of self that exposes their needs. The visibility of the mother and integration of her needs within the child protection plan is critical to achieving the international priority of successfully safeguarding children (United Nations, 1989). The mechanism of mother-centred, empathic support is activated within this context provides the optimum opportunity for mothers to (per)form a destigmatised maternal identity, conducive of safely caring for their infant. Where infants are removed without a plan for reunification, mothers are left with toxic feelings of guilt and shame. Services are typically withdrawn, or the relationship between the mother and the service breaks down resulting in the disengagement of mothers (Kenny & Barrington, 2018). In situations where the mother perceived that their relationship with their infant is not recognised, her loss is not acknowledged, and the mother herself is not valued, she may be at increased risk of developing disfranchised grief (Doka, 2002; Grant et al., 2023). This creates an even more challenging context for future pregnancy. Ambiguous loss due to physical separation (Boss, 2016) and where the separation is seen to be in the best interests of the child (Baum & Negbi, 2013), may compound stigmatising ‘bad mother’ identities and interrupt the grieving process (Doka, 2002). The stigmatisation of mothers at risk of repeat care proceedings is saturated with over simplistic binaries of what it is to be a ‘good’ or a ‘bad’ mother (Whittaker et al., 2020). Such stigmatising identities can trap mothers in cycles of grief and hopelessness, wherein they are subject to repetitive assessments of their parental deficits and experience recurrent patterns of loss. However, there are many ways in which context relevant ‘good mother’ identities can be enacted. Where a safe and supportive relationship with a mother endures beyond infant removal, the involvement of mothers in processes and activities relating to the care of their infant may be encouraged within this key transitionary period. Involvement in these activities may enable the mother to re-negotiate their stigmatised identity and perform tasks compatible with being a ‘good mother’ (from spending time with their infant, feeding and bathing to contributing to decisions about their care and providing content for their life story book). Participation in these tasks may also facilitate sense-making (where mothers make sense of why their infant has been removed) and meaning reconstruction (where the mothers are able to reconstruct care entry as being ‘good’ for their infant). Loss-orientated and restoration-orientated coping of this kind, has been found to facilitate adaptive grief in parents bereaved by death (Stevenson et al., 2017) and is likely to be a necessary component of interventions to enhance the mental health and welling of mothers who experience loss following removal.

### 4.1 Practice implications

The findings of our study have important implications for policy and practice when intervening with mothers at risk of (repeat) care proceedings. Firstly, multi-agency safeguarding teams should look to separate supportive functions from safeguarding functions within the child protection plan. This approach may best enable mothers to experience the level of safety and trust necessary for them to accept and address identified risk. Within the current study, the functions of support have been provided by a national charity external to the child protection department, creating a clear separation at an organisational level. This is an approach that has been provided in other service models addressing sensitive issues within the context of child protection (Cox et al., 2020; Harwin et al., 2014; McCracken et al., 2017; Rodger et al., 2020). More recently, a number of child protection departments in the UK are beginning to bring such services ‘in-house’, seeking to provide additional and targeted support for pregnant women at risk of repeat care proceedings. These specialist pre-birth child protection teams often include social workers and family support workers, which may offer an opportunity for some separation of functions at an individual practitioner level. However, it is unclear whether the findings of ‘what works’ in the Startwell intervention would transfer fully into these models. Further theory-driven evaluations of these services are needed to examine whether it is possible to provide both support and safeguarding within the same, statutory team. Moreover, there is an increasing recognition of the important role fathers play in child and family outcomes (McGovern et al., 2023) and calls for father inclusive child protection practice (Philip et al., 2018). Whilst it seems plausible that fathers may be similarly impacted by the threat of safeguarding, fatherhood provides a different context for intervention which should be considered in future research.

Secondly, there should be a trauma-informed focus upon the mother's needs within plans which seek to safeguard the infant. There should be a recognition and response to the impact of trauma upon mothers, rather than placing emphasis upon a deficit-orientated assessment of risk posed to the infant. Such an approach is most likely to prevent care entry and where it is not safe to do so, promote decisive action. Our study found that mothers who feel that their needs are recognised are more likely to discuss the difficulties they experienced and moreover, were receptive to advice, guidance, and challenge to address the risks identified in the child protection plan. This is an important finding in light of the parental resistance and avoidant relationships which are commonly evident in child protection practice (Ferguson et al., 2021; Forrester et al., 2012; Smithson & Gibson, 2017). However, our study also found that mothers with mild to moderate learning difficulties may benefit less from the support offered. It is likely that adaptations will be needed to better meet the needs of these mothers (Atkin and Kroese, 2022; Collings et al., 2018).

Further, mothers should receive on-going support from a professional after infant removal. This support should be provided from a practitioner whose role is focused upon the mother's wellbeing and not safeguarding the infant. Without such support, the impact of child removal upon mothers has been found to deepen (Broadhurst & Mason, 2020; Kenny & Barrington, 2018), and the task of responding to the requirements of the children protection plan typically becomes overwhelming (Fuentes-Peláez et al., 2016; Kenny & Barrington, 2018). This provision of on-going support to the mother presents the maximum opportunity for mothers to work towards reunification. Where reunification is not in the best interests of the infant, the mother should be supported to participate in activities which allow them to demonstrate care for their infant (such as have time with their infant after birth, participate in meetings where their infants care is discussed and contribute to their infant's life story book) and begin to grieve the loss of their infant.

Finally, there is an increasing recognition of the important role fathers play in child and family outcomes (McGovern et al., 2023) and calls for father inclusive child protection practice (Philip et al., 2018). Whilst it seems plausible that fathers may be similarly impacted by the threat of safeguarding, and require compassionate support to grieve the loss of their infant, fatherhood provides a different context for intervention which should be considered in future research.

### 4.2 Strengths and limitations

To our knowledge, this study is the first to utilise realist methodology to provide explanatory evidence of what works to support the mental health and wellbeing of mothers at risk of recurrent care proceedings, and to prevent care entry of their infants. The result is a middle range theory specific enough to clearly explain the phenomenon, and general enough to apply across cases of the same type, capable of informing care for mothers at risk of repeat care proceedings. Further, our use of quantitative data to test and refine our programme theory is novel within realist methodology (Hawkins, 2016) and is further strengthened by rigorous methods and comparative design. The study does have a number of limitations however. Our quantitative data collection occurred within a relatively short implementation window and the restricted capacity of the service. This resulted in a small sample size. Whilst the use of administrative data represented an ethical, low burden approach to data collection, our ability to test context-mechanism-outcome configurations was limited to the data available. We were able to assess care status within the first 12 months of life however children may have continued to be in care/re-entered care beyond this timepoint. Our qualitative study engaged mothers who had avoided care, experienced reunification following a period of care and whose children had been removed and were not reunified. Whilst this is likely to have supported a wider range of experiences of Startwell to be captured, our recruitment strategy was not able to recruit mothers who were referred to Startwell but did not engage. It is possible that these mothers experienced contexts which were different to those mothers who we recruited, and therefore may limit the generalisability of our findings. Whilst the scope of our study necessarily focused upon mothers as the recipients of the intervention, fathers make an important contribution to both the risk and resilience present within family life (Philip et al., 2018). The absence of fathers in our study therefore limits the ability of our study to make recommendations for practice in this area.

## 5 Conclusion

To support the mental health and wellbeing of mothers at risk of repeat care proceedings and prevent care entry, a mother-centred and empathic approach is necessary. This destigmatising approach is likely to lead to more accurate and decisive assessment, increase reunification and support mothers to grieve following infant removal (Table 6).

**Table 6:** Logistical regressions.

**Logistical regression 1: Being born into care**				
**Coefficients**	**Odds ratio (adjusted)**	**Confidence Interval**	**p value**	
Category of abuse	Neglect (baseline)	1.000	–	–
	Emotional	0.454	0.164 to 1.258	0.129
	Physical	2.015	0.472 to 8.611	0.344
	Sexual	3.240	0.165 to 63.322	0.438
IMD Decile	1 (baseline)	1.000	–	–
	2	0.429	0.168 to 1.091	0.076
	3+	1.888	0.756 to 4.717	0.174
Known more than 12 weeks before birth^a^	2.090	1.020 to 4.283	0.044	
Startwell	2.409	1.157 to 5.014	0.019	
Constant	0.498	0.244 to 1.016	0.055	
***Logistic regression 2: Being in care at* 6 months *of age***				
**Coefficients**	**Odds ratio (adjusted)**	**Confidence Interval**	**p value**	
Category of abuse	Neglect (baseline)	1	–	–
	Emotional	1.232	0.326 to 4.663	0.758
	Physical	0.18	0.019 to 1.667	0.131
	Sexual	0.546	0.014 to 21.772	0.748
IMD Decile	1 (baseline)	1	–	–
	2	1.116	0.292 to 4.270	0.873
	3+	1.387	0.368 to 5.220	0.629
Known more than 12 weeks before birth	0.213	0.062 to 0.734	0.014	
Born into care	69.269	17.410 to 275.600	0	
Startwell intervention	0.503	0.158 to 1.601	0.245	
Constant	0.427	0.162 to 1.125	0.085	
***Logistic regression 3: Being in care at* 12 months *of age***				
**Coefficients**	**Odds ratio (adjusted)**	**Confidence Interval**	**p value**	
Category of abuse	Neglect (baseline)	1.000	–	–
	Emotional	0.739	0.240 to 2.281	0.599
	Physical	0.380	0.064 to 2.257	0.287
	Sexual	1 (empty)	–	–
IMD Decile	1 (baseline)	1.000	–	–
	2	1.048	0.326 to 3.361	0.938
	3+	0.757	0.257 to 2.229	0.613
Known more than 12 weeks before birth	0.615	0.251 to 1.506	0.287	
Born into care	10.685	3.963 to 28.806	0.000	
Startwell	0.377	0.143 to 0.994	0.049	
*Logistic regression 4: Being in care at*12 months*; Startwell & born into care as a combined coefficient*				
**Coefficients**	**Odds ratio (adjusted)**	**Confidence Interval**	**p value**	
Category of abuse	Neglect (baseline)	1.000	–	–
	Emotional	0.758	0.242 to 2.379	0.635
	Physical	0.340	0.054 to 2.154	0.252
	Sexual	1 (empty)	–	–
IMD Decile	1 (baseline)	1.000	–	–
	2	0.984	0.303 to 3.198	0.978
	3+	0.758	0.255 to 2.254	0.618
Known more than 12 weeks before birth	0.600	0.243 to 1.480	0.267	
Startwell and born into care	No Startwell, not born in care (baseline)	1.000	–	–
	No Startwell, born in care	14.820	4.325 to 50.784	0.000
	Startwell, not born in care	0.670	0.155 to 2.905	0.593
	Startwell, born in care	3.951	1.293 to 12.014	0.016
Constant	0.377	0.148 to 0.958	0.040	

a We theorised that the quality of the relationship between the practitioner and the mother may be an important mechanism within a context where mistrust of services is prevalent. We selected a ‘more than 12 weeks before birth’ to test this theory within our logistical analysis.

## CRediT authorship contribution statement

**R. McGovern:** Writing – original draft, Supervision, Methodology, Funding acquisition, Formal analysis. **E. Geijer-Simpson:** Writing – review & editing, Investigation, Formal analysis. **S. O'Keeffe:** Writing – review & editing, Investigation, Formal analysis. **W. McGovern:** Writing – review & editing, Investigation. **S. McCarthy:** Writing – review & editing, Formal analysis. **M. Lhussier:** Writing – review & editing, Methodology. **A. Bate:** Writing – review & editing, Supervision, Formal analysis.

## Declaration of competing interest

The authors declare that they have no known competing financial interests or personal relationships that could have appeared to influence the work reported in this paper.
